# MicroRNAs in aging male reproduction

**DOI:** 10.18632/aging.204003

**Published:** 2022-04-05

**Authors:** Lu Zheng, Jinzhao Ma, Bing Yao

**Affiliations:** 1 Center of Reproductive Medicine, Nanjing Jinling Hospital, Clinical School of Medical College, Nanjing University, Nanjing 210002, Jiangsu, China

**Keywords:** aging, miRNAs, reproduction

Owing to societal pressures, increased life expectancy, assisted reproduction techniques (ART) and the use of modern contraception, more and more couples are postponing their plans for pregnancy in many countries. Advanced maternal age has been well established as one of the major risk factors for poor reproductive outcomes. However, the influence of paternal age on reproduction is incompletely understood.

Advanced paternal age (APA) tends to be associated with a decline in semen quality. A systematic review using data from 90 studies (93,839 subjects) indicated that semen volume, percentage motility, progressive motility, normal morphology and unfragmented cells declined with age significantly [1]. Inferior sperm parameters are usually connected with undesirable embryonic development and poor pregnancy outcomes.

The mechanisms responsible for age-dependent patterns of decline in semen traits are not fully comprehended, but the damage from reactive oxygen species (ROS) is thought to be an important contributor [1]. ROS are produced in the mitochondria, the intracellular energy metabolism factory, and their abnormal increase usually indicates mitochondrial dysfunction. Increased ROS are correlated with decreased sperm motility and accumulated DNA fragmentation at both the nuclear and mitochondrial levels, which in turn exacerbates the sperm dysfunction and abnormalities [2]. Recently, several studies have demonstrated that miRNAs, encoded by the nuclear genome or mitochondrial genome, not only regulate nuclear genome encoding mitochondria-related proteins, but also could translocate into the mitochondria and regulate mitochondrial genome expression [3]. Zhou and colleagues discovered that miR-151a-5p was significantly increased in severe asthenozoospermia cases compared with healthy controls and miR-151a-5p may participate in the regulation of cellular respiration and ATP production through targeting Cytochrome b [4].

In order to investigate the expression patterns of advanced age on reproduction, our group previously performed high-throughput sequencing of small RNAs in sperm, oocytes, and embryos of aged and young mice. Excluding differentially expressed miRNAs in oocytes, and overlapping the specific miRNAs in sperm with the differentially expressed miRNAs in embryos, we obtained 33 miRNAs that might contain the contributor of embryo development from the sperm of aging males [5]. Meanwhile, some of these miRNAs were associated with mitochondria, including miR-574, miR-128, let-7b, miR-24, and miR-125a. In the previous study of our group, we found that miR-574 was upregulated in the sperm of aging males and was related to poor sperm motility as an adverse predictor. MiR-574 suppresses the mitochondrial function and reduces cellular ATP production by directly targeting mt-ND5 [5]. DNA damage, mainly due to oxidative stress, is a major cause of defective sperm function [6]. Recently our group demonstrates that miR-125a-5p suppresses mitochondrial function and increases cellular DNA damage by targeting Rbm38 and activating the p53 damage response pathway [7]. Kim and colleagues observed that the miR-125 family was an important regulator of the expression and maintenance of maternal effect genes during early embryonic development. They found that microinjection of miR-125 family members would suppress the expression of Sebox and Lin28a and impair early embryogenesis, resulting in the arrest of embryogenesis at the two-cell stage [8]. However, we found that miR-125a-5p induced a developmental delay at specific morula/blastocyst stages in a p21-dependent manner in the aging model [7]. These results indicate that abnormal increased miRNAs in sperm could induce mitochondrial dysfunction, which reduced ATP production through damage respiratory chain and eventually lead to sperm abnormalities. Meanwhile, these studies suggest that abnormal increased miRNAs in sperm might modulate sperm function through a DNA damage response pathway and perturb early embryonic development. The cut-off for APA used in our studies was over 40 years old at the time of conception and further studies should be conducted with more stratification by age and on extensive semen samples. Subsequent construction of knockout mice is also needed to further validate the effect of miRNAs on fertility and offspring in aging men.

**Figure 1:**
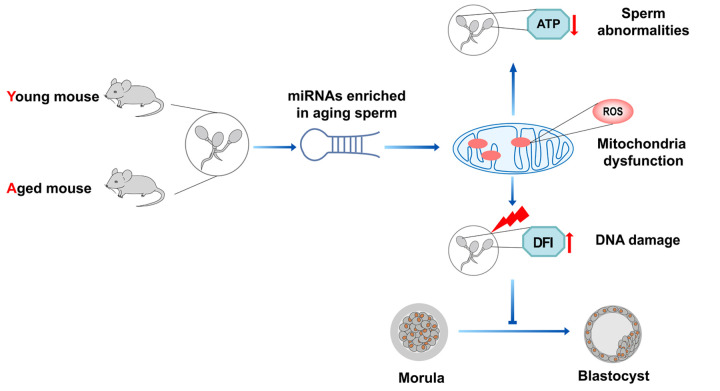
**MicroRNAs in aging male reproduction.** Abnormal increased miRNAs in aging sperm might induce mitochondrial dysfunction, reduce ATP production, perturb early embryonic development.

Collectively, miRNAs play an important role in the regulation of sperm function and embryonic development in aging males, which possibly refer to reactive oxygen species producing in the mitochondria and consequent DNA damage (Figure 1).
